# PatCen: A blockchain-based patient-centric mechanism for the granular access control of infectious disease-related test records

**DOI:** 10.1371/journal.pone.0310407

**Published:** 2024-09-18

**Authors:** Bello Musa Yakubu, Syeda Mahera Ali, Majid Iqbal Khan, Pattarasinee Bhattarakosol

**Affiliations:** 1 Department of Mathematics and Computer Science, Faculty of Science, Chulalongkorn University, Bangkok, Thailand; 2 Department of Computer Science, COMSATS University Islamabad, Islamabad, Pakistan; Jazan University, SAUDI ARABIA

## Abstract

The recent global outbreaks of infectious diseases such as COVID-19, yellow fever, and Ebola have highlighted the critical need for robust health data management systems that can rapidly adapt to and mitigate public health emergencies. In contrast to traditional systems, this study introduces an innovative blockchain-based Electronic Health Record (EHR) access control mechanism that effectively safeguards patient data integrity and privacy. The proposed approach uniquely integrates granular data access control mechanism within a blockchain framework, ensuring that patient data is only accessible to explicitly authorized users and thereby enhancing patient consent and privacy. This system addresses key challenges in healthcare data management, including preventing unauthorized access and overcoming the inefficiencies inherent in traditional access mechanisms. Since the latency is a sensitive factor in healthcare data management, the simulations of the proposed model reveal substantial improvements over existing benchmarks in terms of reduced computing overhead, increased throughput, minimized latency, and strengthened overall security. By demonstrating these advantages, the study contributes significantly to the evolution of health data management, offering a scalable, secure solution that prioritizes patient autonomy and privacy in an increasingly digital healthcare landscape.

## Introduction

In the past, infectious diseases such as yellow fever, Ebola, smallpox, monkeypox, and influenza were among the most feared epidemics. Nowadays, new infections like COVID-19 continue to emerge, while many old epidemics persist, posing global challenges. The rapid spread of infections, exemplified by the COVID-19 pandemic, demonstrates how a new disease can traverse continents within days or weeks [1, 2]. To curb the spread of such diseases, many countries have resorted to strategies such as lockdowns, albeit at the cost of disrupting economic growth [3, 4]. Infectious diseases disproportionately affect vulnerable populations, including those with existing health conditions, children, and individuals over 60 [4–6]. Each infectious disease presents a variety of symptoms, ranging from cough, fever, and fatigue to more severe complications like lung damage, and sensory impairments [4].

One of the most concerning aspects of infectious diseases is the potential for asymptomatic transmission, wherein carriers may spread the disease without exhibiting symptoms [4, 7]. Therefore, infected individuals are required to self-isolate for at least 14 days, the typical incubation period for many diseases, during which they may unknowingly transmit the virus to others [8]. Many countries and organizations have subsequently recommended widespread testing to prevent the spread of diseases. However, publication delays caused by intermediaries and inconsistencies in action plans may thwart effective response strategies.

Recently, researchers have proposed various techniques to address these challenges, such as storing results data in a blockchain public ledger [9, 10]. This approach allows government agencies and organizations to access patient data for infection monitoring, subject to patient consent. This purpose has utilized two types of blockchains: permissioned [11–15] and permissionless [16–20]. Permissioned blockchains are private networks with known users, managed by centralized organizations, while permissionless blockchains operate on peer-to-peer networks and are open to the public [11, 16].

Despite their potential, permissioned blockchains face challenges such as data confidentiality and scalability issues, whereas permissionless blockchains offer tamper-resistant storage and transfer of information. Techniques such as [12, 21–24] have proposed various methods to enhance health record access control, aiming to address privacy risks associated with unauthorized access to patient data. However, they fall short in several areas: they lack mechanisms for privilege designation and authorization, infringe on patient rights by omitting consent requirements, and inadvertently expose sensitive patient data to unauthorized entities [4–6]. These shortcomings not only compromise data privacy and security but also hinder the effective deployment of health interventions during crises [21, 23].

This study presents a blockchain-based approach utilizing self-triggering smart contracts to establish trust and prevent fraud in healthcare systems. By leveraging digital medical credentials and permissioned blockchain technologies, the model facilitates reliable and timely reporting of infectious diseases. Patients can grant access to authorized organizations, verified through unique identification, to support response strategies against disease spread. This approach ensures data integrity and patient privacy while enabling efficient information sharing among stakeholders.

Furthermore, our study illuminates the critical role of blockchain in enhancing EHR access control, offering a detailed examination of its potential to resolve prevalent issues related to data privacy, scalability, and unauthorized access. This work significantly contributes to the discourse on digital health record management and patient empowerment in the era of blockchain technology by presenting a comprehensive security analysis and leveraging game theory to evaluate the proposed model’s robustness.

This article makes several pivotal contributions to the field of healthcare data management and security, leveraging the power of blockchain technology. An enhanced presentation of these contributions is provided below:

- Innovative Access Control Framework: At the heart of this research is the creation and deployment of an innovative access control framework tailored for the healthcare sector. Building upon the robust functionalities of blockchain technology, this framework establishes a new benchmark for secure and efficient data management in healthcare systems.
- Empowering Patients with Data Control: Our study significantly advances patient empowerment by providing them with clear control over their medical records. By enabling informed decision-making, our approach not only fortifies data privacy but also adapts to diverse data access requirements. The novel paradigm it introduces enhances data privacy safeguards, fine-tunes access privileges, and adeptly addresses scalability challenges, all while upholding the sanctity of patient privacy.
- Synergy Between Patient Empowerment and E-Health Services: This study further explores and elucidates the symbiotic relationship between patient empowerment and the efficacy of electronic health (e-health) services. It demonstrates how blockchain technology’s inherent strengths can amplify the benefits of e-health services, thereby fostering a more secure, efficient, and user-centric healthcare ecosystem.
- Rigorous Security Evaluation: Our research conducts an extensive security evaluation of the proposed blockchain-based solution using Decisional Bilinear Diffie-Hellman (DBDH) game theory. Additionally, we conduct a comprehensive security analysis using the Real-Or-Random (ROR) game theory approach to meticulously assess the security features of the session key mechanism. These studies validate the proposed solution’s robustness against various security threats, ensuring a secure and trustworthy framework for healthcare data management.

Through these contributions, our study not only addresses critical gaps in current healthcare data management practices, but also lays the groundwork for future advancements in the secure and effective use of blockchain technology in healthcare and beyond.

The rest of this paper is structured to build upon the foundation laid in this introduction, starting with a detailed literature review that contextualizes our research within the broader field. Subsequent sections delve into the system model, elucidate the proposed PatCen model, offer a thorough security analysis, and present empirical findings from our evaluations, leading to a concluding discussion that highlights key insights and future directions for research in this vital area.

## Literature review

In recent years, several academics have dedicated their efforts to examining the various obstacles and possibilities associated with the integration of blockchain technology into e-health systems [25–27]. This line of inquiry has also included the identification of prospective avenues for further investigation, with a particular focus on the management of patient data access [28]. The concept of EMRs and their prototype, referred to as “MedRec”, was first proposed by Azaria et al. [29] and Hongwei et al. [30] using blockchain-based management framework. Similarly, a blockchain-based e-healthcare system was introduced by [23], which interacts with wireless body area networks using Hyperledger Fabric, achieving good performance in terms of hardware utilization, security, and system stability. However, these frameworks [29, 30] are associated with scalability constraints and can only accommodate a maximum of 51 nodes. To address this issue, a Hyperledger-based e-health technique was introduced in [15, 23] to increase the reliability and stability of e-health services, such as automating healthcare record sharing. However, the scalability issue is inevitable, thus, it is worth noting that these frameworks [15, 23, 29, 30] see medical facilities as the focal point for researching security and productivity challenges, while patients’ privileges are ignored [31].

The significance of allowing patients to grant access to their own medical data was stressed and discussed in [32]. As a result, several works, like [33–36], proposed a patient-centric healthcare data management scheme for privacy preservation. While [37, 38], talked about patient-driven healthcare interoperability and showed how smart contracts can protect the privacy of electronic health records, the authors of [39] used smart contracts to set up an e-health framework that lets a doctor access patient data through system notifications for real-time monitoring. To make hospitals’ records more accessible, [40] proposed a patient-centric model based on smart contracts; however, no robust trial protocol is provided. Correspondingly, [41, 42] proposed a blockchain-based medical data collection scheme and a mixed blockchain-edge architecture, respectively. Similarly, the authors in [43] proposed a blockchain-based network for smart healthcare systems called “S2HS,” in which doctors or practitioners can access data only if patients want to share their medical data. Moreover, [44, 45] used blockchain technologies for healthcare management, allowing the emergency department to view patient medical records in an emergency without the patient’s permission. The various works described above are patient-centric, meaning that anyone who accesses medical data must obtain patient consent. Patients’ rights are undoubtedly well-protected, but this approach also constrains the practicality of e-health services, raising privacy and security concerns like high latency, storage costs, and a single point of failure [46, 47].

Numerous access control approaches for health records have been suggested in the literature [12, 21, 23], with the aim of enhancing security. However, it has been observed that in real-world scenarios, unqualified or unauthorized third parties may gain access to the transaction content of approved third parties, therefore compromising the privacy of patient data [22, 48]. The problem at hand is exacerbated by the use of security techniques that are deemed ineffective, such as public session key security and the existence of transparency concerns within blockchain technology [10, 22]. In current EHR systems, it is common for third parties to use public keys or Ethereum addresses. This practice, however, poses a risk as it might potentially reveal the real identities of these third parties and thereby expose patients’ confidential information to unauthorized individuals. The aforementioned scenario has the potential to give rise to dangers connected to anonymity, hence rendering patients’ sensitive information susceptible to illegal access [10, 22, 48].

To revolutionize smart healthcare record systems by ensuring patients’ rights are well secured and providing real-time services, with improved privacy and security, authors in [49] have proposed a technique termed “Bloccess”, a fine-grained access control framework based on the consortium blockchain. By leveraging blockchain technology, they formulate a set of protocols to enforce a tamper-proof access control mechanism in untrustworthy distributed environments. Similarly, the authors of [50, 51] propose a patient-driven blockchain-based architecture that provides decentralized EHR and smart-contract-based service automation without compromising system security and privacy. In a similar vein, the authors in [47] put out a proposition that enables patients to bestow and withdraw access privileges, while also safeguarding the privacy of healthcare institutions and practitioners. Cloud storage is used to store sensor data, while blockchain is used to maintain access control and session records. The solution employed a data-driven authentication and secure communication protocol, utilizing smart contracts to regulate interactions between the cloud, patients, and healthcare professionals. However, these approaches are susceptible to high latency, high throughput, high computational overhead, and availability issues. Similarly, the approaches may lead to external or internal unauthorized access to sensitive EHR attributes for malicious purposes, patient privacy issues and privilege exploitation [27, 52].

The authors of [53] present a patient-driven approach, known as the triple subject purpose-based access control (TS-PBAC) model, for secure and privacy-preserving IoMT access control, to address issues related to system performance, privacy, and unauthorized access to sensitive EHR attributes. They create hierarchical purpose trees (HPT) and policies to ensure that external users are legal. To enhance the privacy of sensitive attributes, they also introduce LDP-based policies and role-based access control schemes in edge computing. They introduce mutual evaluation metrics using blockchain-enabled records to assess data quality at the patient and medical service levels within an open, anonymous network. Likewise, the authors of [54] present another patient-driven approach that considers the InterPlanetary Health Layer and related Internet of Medical Things (IoMT) implementations. The approach’s primary objective is to manage sensitive data while protecting privacy and ensuring data availability. Specifically, without relying on a third party, users can construct their own private network, collaboratively authorize data operations, and administer their privacy settings. However, patient privacy remains susceptible to data intrusions in these approaches, despite the use of blockchain-based security for electronic health records [55]. Similarly, diverse privileges and scalability present complications for access control systems [56].

To address the issues related to security and privacy concerns, the authors in [57] propose a patient-centric blockchain-based self-sovereign identity (SSI) paradigm for a decentralized self-management of data access control (DSMAC) system. This system enables patients to maintain control over their personal information and grant themselves access to their medical records. In emergency situations, DSMAC employs smart contracts for role-based access control policies, as well as decentralized identifiers and verifiable credentials for advanced access control techniques. Similarly, the authors of [58] introduced a patient-driven approach that scrutinizes the prerequisites for a generalized health passport system. They employ agent-oriented modeling (AOM) to create a blockchain-based self-sovereign identity (SSI) system that integrates with the personal health record (PHR), safeguarding end-users’ privacy and empowering them to manage the data they utilize for credential verification. Even though these approaches have strengthened blockchain-based e-health services in many respects, they have not expressly considered patients’ privileges [27]. Table 1 provides a summary of the most similar existing approaches.

**Table 1:** Literature review.

References	Objectives	1	2	3	Limitations
[15, 23, 29, 30]	The blockchain-based management system to handle EMRs	×	×	✓	Third party privileges are not considered, no classification of data.
[32, 33]	Patient’s data access management mechanisms to address the issue of patients’ privileges	×	✓	×	Patients’ privileges are not considered, plus none-patient driven.
[49–51]	Patient’s data access management mechanisms to address the issue of user privileges	×	✓	×	User privileges are considered except the patients’ privileges, none-patient centric.
[53, 54]	Patient’s data access management mechanisms to address the issue of data privacy	×	×	✓	Patient privileges are not considered, including diverse privileges and scalability complications.
[12, 21, 23, 57, 58]	Patient-centric healthcare data access management scheme for privacy preservation.	×	×	✓	Patient centric, Patients’ privileges are not fully considered.
[34–36, 43],	Patient-centric healthcare data management scheme for privacy preservation.	×	✓	×	Patients’ privileges are not considered, including none-patient driven.
[37, 38, 40, 41]	To protect the privacy of electronic health records.	×	×	✓	Third party privileges are not considered, and have no classification of data.
[42, 45]	To view patient medical records in an emergency without the patient’s permission	✓	×	×	Patient centric, patients’ privileges are not fully considered.

**1:** Patients have partial control over their records. **2**: Patients have no control over their records. **3**: Patients have full control over their records.

Several other studies have provided significant perspectives on how blockchain can enhance security, privacy, and efficiency in several fields, which closely aligns with PatCen’s goals. Sharma et al. (2023) [55] introduces a framework that leverages blockchain technology to safeguard privacy in healthcare systems powered by the IoT. The framework offers a decentralized method for organizing medical records. Gaur et al. (2023) [59] emphasizes the need for strong security measures to defend against adversarial attacks in cyber-physical systems, emphasizing the necessity of safeguards to guarantee the integrity of data. Zhang et al. (2023) [60] provide a system for privacy-preserving distant sensing picture identification that uses visual cryptography. This highlights how important it is to protect patient privacy with private medical information. D. Charles (2023) [61] studied a cross-border payment system that demonstrates the versatility and growth potential of blockchain technology, essential for its potential expansion and seamless integration with other systems. A prevalent technological vulnerability found in these models is the intricate nature and demanding processing needs linked to blockchain and cryptography methods. This can result in longer response times and decreased efficiency, making it difficult to deploy these systems on a broad scale [62].

### Problem statements and objectives of the study

The scholarly inquiry within the domain of blockchain-based e-health systems predominantly revolve around two central themes: data access management and privacy preservation. However, there exists an imperative need for the development of a comprehensive access control framework that places paramount importance on patient-centric principles and exhibits adaptability to diverse access scenarios, all while upholding the inherent rights and privileges of the involved patients. Presently, prevailing methodologies often overlook the crucial aspects of data classification, patients’ rights and privileges, and inadequately address the intricate challenges posed by scalability.

### Preliminaries

This section discusses the preliminaries of the overall system model, including the network model, adversary model, system requirements and data classification and its impact on access control in proposed system.

### Network model

**Fig 1:**
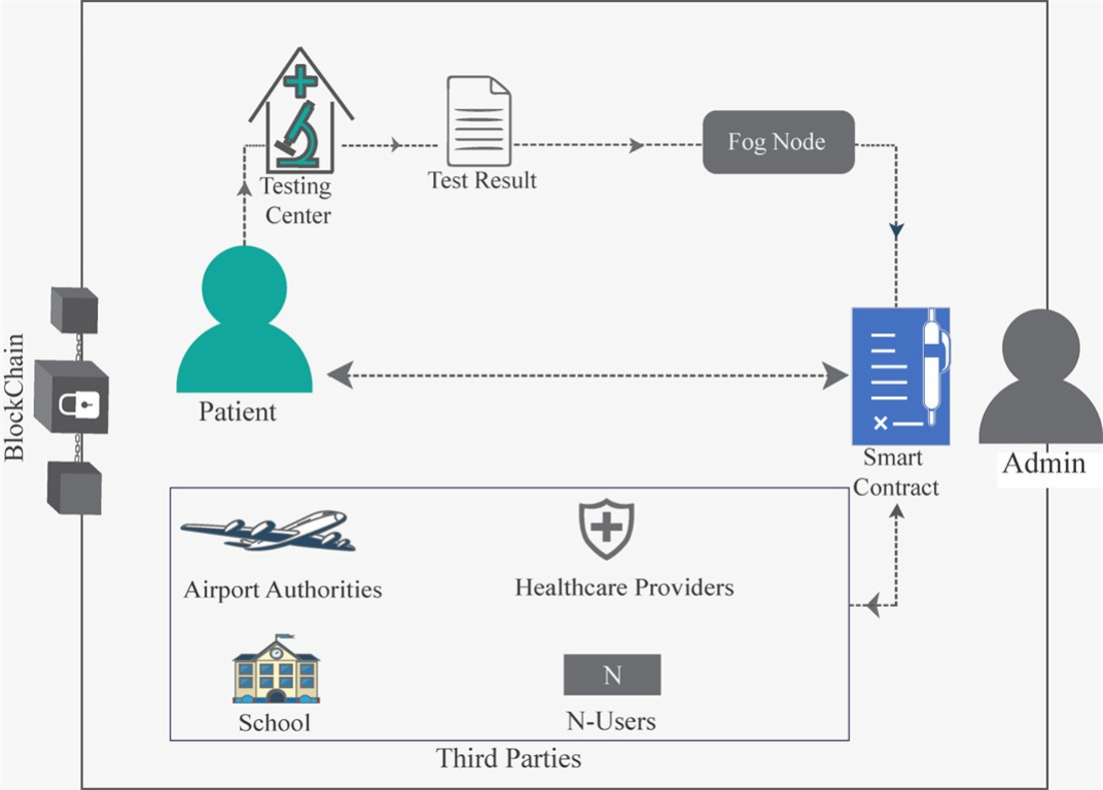
Network model.

The utilization of a decentralized private Ethereum blockchain forms the backbone of our network architecture. Ethereum blockchain, renowned for its robustness, transparency, and smart contract functionalities, provides a secure and immutable platform for our data sharing ecosystem. As depicted in Fig 1, the network comprises four key stakeholders: users (third parties), patient, laboratories, administrator (admin), each possessing distinct Ethereum accounts (EA). The admin plays a pivotal role in system governance by registering stakeholders and overseeing their participation.

The patient, acting as the data owner, initiates the data sharing process by providing samples and relevant information to laboratories. This information may be related to medical, educational, or travel-related data. Meanwhile, laboratories utilize sample processing devices to generate test results. These results as well as the associated relevant information are securely uploaded to the blockchain via fog nodes, ensuring accessibility while maintaining data integrity. Subsequently, access permissions are granted to other users as necessitated, further reinforcing the patient’s control over their data. The data access granting process is facilitated through a mobile application, wherein the patient verifies and grants access permissions to requesting users via the smart contract.

Users, representing third-party entities, seek access to patient records within the system via smart contract based on predefined roles. Access permissions are granted for specified durations, with provisions for extension upon request. Notably, the patient retains ownership of their data and may grant access to authorized users when needed. This dynamic access control mechanism ensures data privacy and security while facilitating seamless information exchange.

By leveraging the Ethereum blockchain, our network guarantees transparency, immutability, and security, thereby establishing a robust foundation for patient-centric data sharing in healthcare and beyond.

### Adversarial model

In this work, we assume that the adversary can conduct a sequence of malicious attacks on the system and may possess the following capabilities:

1. *Direct Data Attacks*: The adversary may attempt to directly attack the patient’s data to gain unauthorized access or steal data stored in the public ledger. This includes techniques such as hacking into the system, exploiting software vulnerabilities, or launching phishing attacks to obtain sensitive information.
2. *Unauthorized Third-Party Attacks*: The adversary may be an unauthorized third party who tries to infiltrate the network to access data. This type of adversary could leverage social engineering, network eavesdropping, or brute force attacks to gain unauthorized access to the blockchain network.
3. *Insider Threats*: The adversary may be an authorized third-party user who seeks to access data beyond their intended scope (e.g., an educational user attempting to access travel-related health information). This insider threat scenario highlights the risk of privilege escalation and the need for strict access controls.
4. *Blockchain Integrity Assumption*: We assume that the blockchain itself is secure and cannot be compromised. This assumption is based on the inherent security properties of blockchain technology, such as cryptographic hashing, decentralized consensus mechanisms, and immutability.

### System requirements

1. The system needs to preserve safe data sharing with legitimate third-party users based on their purpose.
2. The system fails when access permission is given to a legitimate user with a different purpose or when access is given to a non- legitimate user with or without the permission of the patient.

### Data classification and its impact on access control in proposed system

In the proposed system, data classification is a fundamental component designed to protect sensitive healthcare information, specifically focusing on infectious disease test records. This classification system is tailored to the needs of the four key stakeholders involved: patients, laboratories, administrators, and third-party users (e.g., airport authorities, healthcare providers, and educational institutions). The classification of data directly influences how access is controlled, ensuring that each stakeholder can access the appropriate information necessary for their role while maintaining the security and privacy of patient records.

#### Types of data classified

The proposed system classifies infectious disease test records and related data into distinct categories, each with specific access permissions based on the stakeholder’s role:

1. **Patient information:**
  - **Examples:** Patient identifiers, consent records, and historical test results.
  - **Classification impact:** This data is highly sensitive and primarily accessible to the patient and the laboratory that generates the test results. Patients have the authority to control who else can access this information, such as third-party users for specific purposes (e.g., travel clearance). The system ensures that patients can selectively share their information with authorized third parties while maintaining control over their data.
2. **Laboratory data:**
  - **Examples:** Test results, laboratory notes, and diagnostic reports.
  - **Classification impact:** Laboratory data is restricted to the laboratory that generates the test results and the patient. Laboratories are responsible for securely uploading this data to the blockchain, where it is then accessible to the patient. The laboratory data is also made available to third-party users only when the patient grants explicit permission. This classification ensures that test results remain confidential until the patient decides to share them.
3. **Third-party access data:**
  - **Examples:** Data requested by third parties such as airport authorities, healthcare providers, or educational institutions.
  - **Classification impact:** Third-party access data is governed by the permissions set by the patient. For instance, if a patient needs to provide proof of a negative test result for travel purposes, they can grant specific access to the relevant airport authority. The system enforces these permissions through smart contracts, ensuring that third-party users only access the data necessary for their specific use case and only within the scope defined by the patient.
4. **Administrative data:**
  - **Examples:** System logs, user access records, and permission settings.
  - **Classification impact:** Administrative data is primarily accessible to system administrators, who are responsible for managing the blockchain infrastructure, ensuring compliance with regulatory standards, and monitoring the system for any unauthorized access. This data includes logs of all access requests and permissions granted, providing a transparent audit trail. Administrators do not have access to the actual test results or patient identifiers unless explicitly permitted by the patient for system maintenance or legal compliance purposes.

#### Impact on access control

The classification of data within the proposed system directly influences how access control mechanisms are implemented for each stakeholder:

- **Patient-centric control:** Patients are at the centre of the access control process, with the ability to manage who can access their infectious disease test records. The classification ensures that patients can selectively share their data with third parties based on the specific needs of each situation (e.g., traveling, healthcare, education) while retaining overall control over their personal health information.
- **Laboratory-patient confidentiality:** The direct communication channel between laboratories and patients ensures that test results are only shared when necessary. Laboratories upload the results to the blockchain, but the data remains confidential until the patient chooses to share it with a third party. This classification supports strict confidentiality and minimizes the risk of unauthorized access to sensitive health data.
- **Third-party access regulation:** Third-party users, such as airport authorities or healthcare providers, are only granted access to the data that the patient deems necessary. This is enforced through the smart contracts embedded in the blockchain, which ensure that access is both limited in scope and time-bound, preventing misuse or overreach.
- **Administrative oversight:** Administrators have access to system-level data, which allows them to manage the blockchain and enforce compliance without compromising patient privacy. The classification ensures that administrators can perform their duties without accessing sensitive health information unless specifically required for system integrity or legal compliance.

The data classification system in proposed system is tailored to the unique roles and responsibilities of the key stakeholders involved in managing infectious disease test records. By categorizing data based on its relevance and sensitivity to each stakeholder, PatCen ensures that access to healthcare information is strictly controlled, supporting patient privacy while enabling necessary interactions with third-party users. This classification-driven access control mechanism is central to the security and effectiveness of the proposed system, ensuring that sensitive data is handled in a secure, transparent, and patient-centred manner.

### Proposed PatCen model

This section provides a detailed description of the proposed patient centric (PatCen) model’s structure. The model consists of two distinct components: first, the granular data access control mechanism, followed by the second, the attribute-based data confidentiality scheme. These two components are distinctive and considerably contribute to the proposed PatCen model’s security and efficient implementation. The subsequent subsections provide detailed explanations of the procedures involved. Table 2 provides descriptions of some important symbols used in the proposed model.

**Table 2:** Key terms and descriptions.

Symbol	Description	Symbol	Description
RID	Requester ID	*TK*	Encrypted text
***θ***	Validity period	*ϑ*	Other vital information associated with patient or third-party user
***RBK*_*i*_**	User i public key	Ð	Data
***Gateway*_*id*_**	Admin ID	M	Original message
***RType***	Third-party user’s request type	*U*_*y*_	Users (general)
***List*_*i*_**	A given list	*U*_*y1U*_	Third-party user
***IndexList*_*i*_**	A given Index list	*U*_*y2G*_	Gateway (admin) user
***request*_*i*_**	A given request	*U*_*y3P*_	Patient user
***G*′** and ***G*′′**	Multiplicative cyclic groups of prime order ***p***	***A***	Adversary
***h***	The generator of ***G***′	*hf*(.)	Hash function
***m***	A bilinear map function	*K*_*pub*_	Public key
***x*,*y* ∈**	Elements of ***G***′	*M* ***_K_***	Master key
***s*,*t*,*w***	Elements of ℤ*p*	*K*_*prv*_	Private key
***A*_*atr*_ *= {a*_1_*……*,*a_N_}***	A set of attributes	*δ*, *δ’*, *h*_2_, *φ*_1_, *φ*_2_, *σ*, η, *ρ*	Random values
***U*** and ***AS***	Attribute list of users and access structures, respectively	DBDH	Decisional Parallel Bilinear Diffie-Hellman Exponent

### Granular data access control mechanism

This section presents a thorough clarification of the proposed model’s granular data access control mechanism. Employing Ethereum smart contracts, immutable logs, and trusted events, the model achieves its objectives effectively. It facilitates the sharing of patient data with authorized third parties, while significantly reducing the risk of data leakage and unauthorized access. This feature proves advantageous, particularly in identifying infectiously positive patients and helping users avoid contact with them.

Fig 1 illustrates the system diagram for the proposed model, depicting the process flow wherein the patient submits a sample to the testing center. Subsequently, the testing result is published and stored on the blockchain using a fog node. The subsequent operational processes of the system are clarified in the following subsections, providing a comprehensive overview of the system’s functionality and workflow.

#### Initialization and registration

When third-party users, such as those from the education department, require access to patient data within the system, they must undergo a registration process. This involves submitting their credentials, including affiliation and EA. For example, if a third-party user belongs to the education department, they specify their affiliation, triggering the generation of a unique session key known as the Requester ID (RID). The RID serves as evidence of the user’s registration and inclusion in the list of third-party users associated with the relevant sector, ensuring streamlined access control. The RID has a predetermined expiration period (*θ*), ensuring security and access control within the system. This study assumes a sufficient validity period for at least one complete access request cycle, though, the system architecture can be adjusted accordingly.

Algorithm 1 outlines the procedure for registering a third-party user within the system. Initially, the user initiates the process by sending a request message to join the network, accompanied by their public key (*RBK*_*i*_) via the gateway. The validity of this message is verified by checking the freshness of its timestamp, as depicted in line 3 of the algorithm. Upon validation, the user’s *RBK*_*i*_ is mapped to the corresponding administrator (*Gateway*_*id*_) as illustrated in line 5. Consequently, a new RID (*RID*_*i*_) is generated for the third-party user, as depicted in line 6. This RID serves as the key for the third-party user to engage with the network during subsequent interactions related to patient data access.

This streamlined registration and initialization process ensures the secure integration of third-party users into the system, facilitating efficient and controlled access to patient data while upholding data security and confidentiality protocols.

**Algorithm 1** **: Third party user registration**
**Input** *RBK_i_, Gateway_id_, Timestamp*
**Output** *RID_i_*
**Start**
1 ***If*** *Current.Timestamp > Timestamp **Do***
2  ***For*** *all RBK_i_ received **Do***
3   *Map RBK_i_ to Gateway_id_*
4   *RID_i_ = hash (RBK_i_,Gateway_id_)*
5  ***End For***
6  *Return RID_i_*
7 ***End If***
**End**

#### Classification mechanism

The role-based classification mechanism, outlined in Algorithm 2, plays a crucial role in the proposed model’s access control system. Upon receiving an access request, the classification function within the system retrieves the third-party user’s request type (*RType*) and Requester ID (*RID*). This process is facilitated by maintaining a registry that stores and identifies each third-party user’s entry in the system.

Algorithm 2 efficiently categorizes incoming requests based on their types. For each identified request type, the algorithm generates an index list that associates the correct request with its corresponding request number. This systematic approach, as demonstrated in lines 9–12, 13–16, and 17–22 of Algorithm 2, streamlines the process and enhances the system’s responsiveness.

Once third-party users are successfully added to the system, they are classified according to their specific roles or purposes for accessing patient data. This classification ensures that each user is granted access only to data attributes relevant to their designated role. For instance, users affiliated with the education department are authorized to access and retrieve data attributes pertinent to the education sector exclusively. Conversely, access to data attributes outside their designated field is restricted.

Furthermore, certain attributes, such as ID card numbers or infectious disease test results, are designated as accessible to all users. This ensures the availability of critical information to relevant stakeholders while maintaining the integrity and security of sensitive data.

Overall, the role-based classification mechanism, coupled with granular access control, not only enhances data security but also facilitates efficient data management within the proposed model. This approach ensures that access to patient data is regulated, aligning with privacy regulations and promoting responsible data usage practices.

**Algorithm 2** **: Classification**
**Input:** Entire list of requests (*List*_*n*_)
**Output:** Lists for different request types (*List*_*edu*_, *List*_*travel*_, *List*_*health*_), *IndexList*_*i*_
**Start**
1 **Initialize Lists:** Initialize *List*_*edu*_, *List*_*travel*_, and *List*_*health*_ to store requests based on their types.
2 **Begin** Classification:
3  Iterate through **each** request **in** *List*_*n*_.
4  Based **on** the request type (*RType*_*i*_):
5   ***If*** *RType*_*i*_ is Educational ***Then***
6    Add the request to *List*_*edu*_.
7    Count the number of requests and update *IndexList*_*edu*_.
8    Send *IndexList*_*edu*_ to the patient.
9   ***Else If*** *RType*_*i*_ is Traveling ***Then***
10    Add the request to *List*_*travel*_.
11    Count the number of requests and update *IndexList*_*edu*_.
12    Send *IndexList*_*travel*_ to the patient.
13   ***Else*** (*RType*_*i*_ is Health)
14    Add the request to *List*_*health*_.
15    Count the number of requests and update *IndexList*_*health*_.
16    Send *IndexList*_*health*_ to the patient.
17  ***If*** all requests have been processed, **exit** the **loop**.
18 **Stop** Classification: **If** no more requests are received, **stop** the classification process.
**End**

#### Verification, granting access & revoking access

Algorithm 3 serves as the foundation for verifying the legitimacy of a requester within the system framework. It employs a validation mechanism where the *RID*_*i*_ is authenticated against the system’s records. If the *RID*_*i*_ matches the hash generated from the requester’s public key and the gateway ID (as detailed in line 5 of Algorithm 3), the request is deemed valid, indicating that the third-party user is duly registered within the network. Conversely, a mismatch leads to the request being tagged as invalid, signaling an unauthorized attempt to access the system.

**Algorithm 3**: Verification
**Input** *RBK_i_, Gateway_id_, Timestamp*
**Output** *Verification Status*
**Start**
1 *Request received = **True***
2 ***While** Request received = **True***
3  ***If** RID_i_ = hash (RBK_i_,Gateway_id_)* **Then**
4   *Return "Valid RID_i_"*
5  ***Else***
6   ***Return*** *"Invalid RID_i_"*
7  ***End If***
8 *Request received = **False** // Assuming condition to exit loop*
**End**

Building upon the verification process, Algorithm 4 outlines the protocol for authorizing access to data owned by the patient. The crux of this algorithm lies in matching the *RID* against the index list (*IndexList*_*i*_), as elucidated in its operational steps. A successful match validates the request, affirming that it originates from a recognized third-party user. Should the *RID* fail to find a corresponding entry in the index list, the request is invalidated (referenced in the conditional checks of the algorithm). Following the validation of a request, the algorithm further evaluates if the request falls within the designated access timeframe. Access is consequently sanctioned only upon satisfying both the validation of the request and the temporal constraints.

**Algorithm 4** **: Granting Access**
*Access time = **True***;
***Function*** *GrantAccess*()
**Start**
1 ***For*** *Request_i_ in List_i_ where i can be travel,health or educational*
2  ***If*** *RID_i_∈ request_i_ & request_i_ ∈ IndexList_i_ **Then***
3   ***Return*** *"Valid request for RType_i_,RID_i_*
4  ***Else***
5   ***Return*** *"Invalid request"*
6  ***End If***
7  ***If*** *Access time = **True Then***
8   ***Return*** *"Access granted"*
9  ***Else***
10   ***Return*** *"Access denied due to time restriction"*
11 ***End For***
**End**

Algorithm 5 introduces the capability to revoke previously granted access, enhancing the system’s security and flexibility. The revocation process is initiated based on specific criteria, such as the expiration of access time or the absence of the *RID*_*i*_ in the request queue, thereby terminating the access rights.

**Algorithm 5** **: Revoking access**
**Input:** Access time, *RID*_*i*_, *request*_*i*_
**Output:** Access Termination Status
**Start**
1 ***Function*** *RevokeAccess*()
2  ***If*** *Access time =* ***False Then***
3   ***Return*** *"Terminate access due to time restriction*”
4  ***Else If*** *RID*_*i*_ *not in request*_*i*_ ***Then***
5   ***Return*** *"Terminate access due to invalid request ID"*
6  ***Else***
7   ***Return*** *"Access not appropriate for termination"*
8  ***End If***
**End**

**Fig 2:**
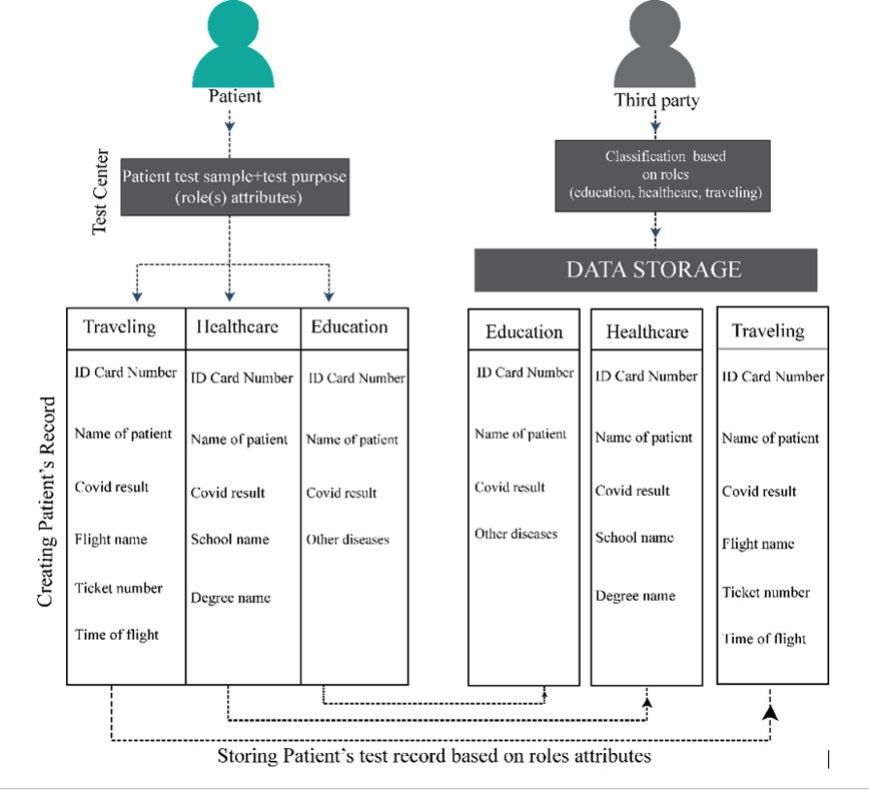
Role based classification.

The interplay of these algorithms ensures a robust mechanism for the classification, verification, and management of access rights within the system. Fig 2 encapsulates this procedural workflow, illustrating the seamless interaction between the third-party users and the data owners. Through the presentation and verification of the *RID*, patients can ascertain the authenticity and authorization of third-party requests. This verification empowers patients to grant access selectively, based on the third-party’s affiliation, thereby safeguarding the privacy and integrity of the patient’s records stored within the data repository.

### Attribute-based data confidentiality scheme

Blockchain storage is often decentralized and transparent, enabling unrestricted public access. Every transaction conducted on the blockchain is transparent and accessible to all participants in the system. For instance, when the system stores user data in the public ledger, transmits it to a third-party user, or verifies its authenticity, it becomes accessible to the participants. Hence, data stored on the blockchain needs to be obscured, and the unencrypted content should only be available to authorized entities.

Thus, this section presents a data confidentiality technique that aims to conceal users’ information while it is being stored or sent by using public key infrastructure (PKI). To achieve this, the following postulations are provided:

***Postulation 1*:** Given two multiplicative cyclic groups ***G***′ and ***G***′′ of prime order ***p***, such that 1D4BD is the generator of ***G***′. If m is a bilinear map function, then bilinear map pairing *m*:***G***′ × ***G***′→***G***′′ will have the following characteristics:
  - $m\left(x^{s},y^{t}\right)=m{\left(x,y\right)}^{st}$, For all *x*,*y* ∈ ***G***′ and $s,t,w\in {\mathbb{Z}}_{p}$ (bilinearity).
  - Bilinear Mapping of *m*: *m* (*x*,*y*) ≠ 1 (non-degeneracy).
  - ***G***′ is considered as a bilinear group if the operation of group in ***G***′ along with the bilinear map m (x,y) *x*,*y* ∈ ***G***′ are effectively computed (computability).
***Postulation 2*:** Let *A*_*atr*_ = {*a*_1_……,*a*_*N*_} be set of attributes, ∋∀*a*_*i*_ ∈ *A*_*atr*_, *U* = {*a*_1_……,*a*_*q*_} and *AS* = {*a*_1_……,*a*_*r*_}, where *U* and *AS* represents attribute list of users and access structures, respectively. Thus, if *U* = *AS*, this implies that *U* satisfies *AS*, i.e., *U*_*i*_ = *AS*_*i*_, where *i* = 1,2,…,*n*.
***Postulation 3*:** Given a Decisional Parallel Bilinear Diffie-Hellman Exponent (DBDH) [63], having an actor *Q* with advantage *T* in solving the DBDH problem in ***G***′, any challenger with random values such as $s,t,w\in {\mathbb{Z}}_{p}$ with a DBDH challenge {(*h*,*h*^*s*^,*h*^*t*^,*h*^*w*^,*W*)} can determine if $W=m{\left(h,h\right)}^{stw}$. Thus, the DBDH postulate holds if no polynomial time process has an insignificant benefit of at least *T* when addressing the decisional DBDH challenge in ***G***′ [64].

The following subsections describes processes involve in developing and sharing of the session key as well as encryption and decryption processes.

**Setup:** In this process a security parameter γ is taken as an input, while the output is given as: Public key (*K*_*pub*_) and master key (*M*_*k*_). From Postulation-1 assumes that some random elements and exponent $h_{2},\varphi_{1},\varphi_{2}\in G^{\prime }$ and ε ∈ ℤ_*p*_ were chosen this implies that *h*_1_ = *h*^*ε*^ and *Z* = *m*(*h*_2_,*h*_1_) can be achieved. Hence, the public is generated as: *K*_*pub*_ = (*φ*_1_,*φ*_2_,*h*,*h*_2_,*h*_1_,*Z*) while the master key is generated as $E_{k}=h_{2}^{\varepsilon }$.

**Key generation:** In this process, the private key (*K*_*prv*_) is generated using the master key (*M****_K_***), public key (*K*_*pub*_) and set of attributes (*AS*). Thus, by choosing a random value, δ ∈ ℤ_*p*_, the private key is generated as: *K*_*prv*_ = *Gen*_*key*_ (*M*_*K*_,*K*_*pub*_,*AS*), and published as *K*_*prv*_: $E_{1}=h^{\delta }$, $E_{2}=h_{2}^{\varepsilon }{\left(\varphi_{1},\prod_{a_{j\in U}}h_{1}^{a_{j}}\right)}^{\delta }$, where *j* = 1,2,…,*n*.

**Encryption:** The inputs to this process include, the public key (*K*_*pub*_), data Ð = {*RID*,ϑ}, and access structure *AS*, thus, the encrypted text, TK, is given as: $\text{TK}=\text{Encrypt}\left(K_{pub},Ð,\text{AS}\right)$, where ϑ represents other vital information associated with patient or third-party user. It initially chooses a random value σ ∈ ℤ_*p*_, then computes *t*′ = *h*^σ^, *t*′′ = Ð. *Z*^σ^, $t^{\prime \prime \prime }={\left(\varphi_{1},\prod_{a_{i\in AS}}h_{1}^{a_{i}}\right)}^{\sigma }$ and $t^{\prime \prime \prime \prime }={\left(h_{2},\varphi_{2}\right)}^{\sigma }$. Hence, the encrypted text is published as: $TK=\left(AS\cdot t^{\prime }\cdot t^{\prime \prime }\cdot t^{\prime \prime \prime }\cdot t^{\prime \prime \prime \prime }\right)$**Decryption:** To generate the data Ð, the inputs to this process will include, the public key (*K*_*pub*_), the private *K*_*prv*_, and the encrypted text TK: $Ð=Decrypt\left(K_{pub},TK,K_{prv}\right)$. In this method, the encrypted text TK can only be deciphered by users who acquires the list of attributes *U* that satisfy the list of access structure *AS*. That is to say, if *U* satisfies *AS*, then the original message (M) can be recovered from the encrypted text, TK, as given in Eq 1 below:

$$Ð=\frac{m\left(E_{1},t^{\prime \prime \prime }\right)\cdot t^{\prime \prime }}{m\left(t^{\prime },E_{2}\right)}=Ð\cdot \frac{m\left[{\left(h_{1},h_{2}\right)}^{\sigma }\cdot {\left(\varphi_{1}\Pi_{a_{i\in U}}h_{1}^{a_{i}},h\right)}^{\delta \sigma }\right]}{\left.m\left[\left(\hbar_{,h_{2}}^{\varepsilon }\right)\cdot {\left(\varphi_{1}\Pi_{a_{j\in AS}}h_{1j}^{a\delta }\right)}^{\delta },h^{\sigma }\right)\right]}$$ (1)

### Security analysis

This section provides evidence that the proposed scheme is secure using game theory approaches. The section begins by demonstrating that the granular data access control mechanism is protected under the real-or-random (ROR) model. The second part demonstrate that the attribute-based data confidentiality scheme is secure against an adversary who poses a significant threat to decisional DBDH.

### Security proof for a granular data access control mechanism using ROR model

Each third party is classified as qualified or unqualified for data access based on their responsibilities in the proposed model. The patient uses the third party’s session key to determine if the third party belongs to the role that allows data access, and then decides whether to grant or deny the third party’s request. From Fig 1, the proposed system *S* has considered three users *U*_*y*_, which are third-party *U*_*y*1*U*_, gateway (admin) *U*_*y*2*G*_, Patient *U*_*y*3*P*_. These are instances y1, y2 and y3 for U, G and P. The adversary ***Z*** can use the session key from the pool of session keys to pretend to be a qualified third party and tries to access the patients’ data by executing various functions in the game model, these includes:

- The adversary may run the function $E\left(U_{y1U},U_{y2G},U_{y3P}\right)$ to eavesdrop on the transmitted messages over a public channel between U, G, and P.
- The adversary may execute the function *J*(*U*_*y*1*U*_) to extract sensitive data from the user’s device *U*.
- The adversary may run the function *K*(*U*_*y*_) to reveal the current session key between *U*_*y*1*U*_ and *U*_*y*3*P*_. If the adversary is not able to expose the session key between *U*__*y*1*U*_ and *U*_*y*3*P*_ using the *K*(*U*_*y*_) function, then the session key is secure.
- The adversary may execute the function *L*(*U*_*y*_ *and M*), to send the message M to (*U*_*y*_) and receive a response message.
- Before the game begins, the adversary executes a function *T*(*U*_*y*_) to get a fair coin *f*_*c*_ throw, with the outcome known only to ***Z***. ***Z*** makes a judgment on the test inquiry based on this outcome. If ***Z*** runs the function and the session key is not from the pool and is new, then *U*_*y*_ returns the session key for *f*_*c*_ = 1 or a random number for *f*_*c*_ = 0. Otherwise, it will return null (Ʇ).

Following the test function’s execution on *U*_*y*_, ***Z*** must differentiate the result value. ***Z*** examines the random bit fc’s reliability using the result of the test function. When the guessed bit $f_{c}^{\prime }=f_{c}$,***Z*** wins the game. Furthermore, each member of the system has access to a collision-resistant cryptographic one-way hash function *hf*(.). Similarly, *hf*(.) is considered as a random oracle in the proposed paradigm, Hash. Using Zipf’s law model [65, 66], the following theorem is proposed:

**Theorem 1.** Given that ***Z*** is capable of breaching the proposed model’s session key security, and the benefit of ***Z*** running in polynomial time is denoted by *ADV****Z***. Then,

$$ADVA\leq \;\frac{a_{h}^{2}f}{\left|Hash\right|}+2\{B.a_{send}^{u}\}$$ (2)

where *a*_*hf*_ is the number of hash values, |hash| is the volume of the hash function *hf* (.), and *a*_*send*_ is the number of send functions. In addition, *B and u* are the Zipf’s parameters.

***Proof*:** We demonstrate the security of the session key using a series of the proposed games *KM*_*I*_, where *I* ∈ [0,3]. *Vict*_*AI*_ denotes the case in which ***Z*** wins *KM*_*I*_, by correctly predicting the random bit *f*_*c*_. *Ur*[*Vict*_*A*_,*KM*_*I*_] denotes the advantage of ***Z*** winning the game *KM*_*I*_. Each game is described in detail below.

***KM*_0_:** This game enables ***Z*** to conduct a real-world attack against the proposed model. At the start of *KM*_0_, ***Z*** selects a random bit *f*_*c*_. Then, in accordance with this game, Eq 3 is obtained.


$$ADVA=|2Mr\left[Vict_{A,KM_{0}}\right]-1|$$ (3)

***KM*_1_:** ***Z*** executes the function $E\left(U_{y1U},\cdot U_{y2G};U_{y3P}\right)$ in this game and listens on the sent messages. Then, ***Z*** executes *K*(*U*_*y*_) and *T*(*U*_*y*_) functions to verify that the derived session key is real or fake. To derive the session key the ***Z*** should know the identities of U, G and P. As a result, there are no such cases in which ***Z*** boosts ***KM*_1_**’s likelihood of winning. As a result, ***KM*_0_** and ***KM*_1_** are vague, and the following result is obtained in Eq 4.


$$Mr\left[Vict_{A,KM_{1}}\right]=Mr\left[Vict_{A,KM_{0}}\right]$$ (4)

***KM*_2_** In this game, ***Z*** runs hash and send functions to retrieve the session key. By changing conveyed communications, ***Z*** may launch an active attack. All exchanged messages, on the other hand, are created using secret credentials and random numbers and are safeguarded using the one-way hash function *hf*(.). Additionally, ***Z*** makes it difficult to extract secret credentials and random nonces due to the fact that it is a computationally infeasible task according to the feature of *hf*(.). As a consequence of using the birthday paradox [67], we get the following conclusion.


$$|Mr\left[Vict_{A,KM_{2}}\right]-Mr\left[Vict_{A,KM_{1}}\right]|\leq \frac{a_{hf}^{2}}{2|\;Hash\;|}$$ (5)

***KM*_3_** Here, the adversary may try to access the session key by executing the function *J*(*U*_*y1U*_) and can extract private values such as password and username, which are stored in the device of the user. However, ***Z*** has no knowledge of these values and, thus, cannot derive any secrete knowledge further. Additionally, it is computationally infeasible for ***Z*** to estimate both password and username concurrently. In conclusion, ***KM*_2_** and ***KM*_3_** are literally identical. The following result can be reached by using Zipf’s law.


$$|Mr\left[Vict_{A,KM_{3}}\right]-Mr\left[Vict_{A,KM_{2}}\right]|\leq B\cdot q_{send}^{u}$$ (6)

Assuming that all games have been completed, ***Z*** must predict the bit in order to win the game. As a consequence, the following result is obtained.


$$Mr\left[Vict_{A,KM_{3}}\right]=\frac{1}{2}$$ (7)

Using Eqs (1) and (2), the following result, Eq 8.


$$\frac{1}{2}ADVA=|Mr\cdot \left[Vict_{A,KM_{0}}-\frac{1}{2}\right]=\left[Vict_{A,KM_{1}}-\frac{1}{2}\right]|.$$ (8)

Then, Eq (9) is derived using Eqs (7) and (8).


$$\frac{1}{2}ADVA=|Mr\cdot \left[Vict_{A,KM_{1}}-\frac{1}{2}\right]=\left[Vict_{A,KM_{3}}-\frac{1}{2}\right]|.$$ (9)

With Eqs (6), (7), (8) and (9), the following conclusion using the triangle inequality can be obtained.


$$\left.\frac{1}{2}ADVA=|Mr\left[Vict_{A,KM_{1}}\right]-Mr\left[Vict_{A,KM_{3}}\right]\right|\leq \left|Mr\left[Vict_{A,KM_{1}}\right]-Mr\left[Vict_{A,KM_{2}}\right]\right|+$$

$$\left|Mr\left[Vict_{A_{A,KM_{2}}}\right]-Mr\left[Vict_{A,KM_{3}}\right]\right|\leq \frac{a_{hf}^{2}}{2|\;Hash\;|}+\;B.\;a_{send}^{u}$$ (10)

By multiplying both sides of Eq (10) with 2, the required results can be displayed as follow:

$$ADVA\leq \frac{a_{hf}^{2}}{|\;Hash\;|}+2\left\{B\cdot a_{send}^{u}\right\}$$ (11)

Hence, theorem is proved.

### Security proof for attribute-based data confidentiality scheme

**Theorem 2:** If the decisional DBDH challenge can be thwarted, an adversary-using actor may be able to circumvent it with a significant benefit.

**Proof:** Assuming that an adversary ***A*** with random values *s*,*t*,*w* ∈ ℤ_*p*_ with a DBDH challenge {(*h*,*h*^*s*^,*h*^*t*^,*h*^*w*^,*W*)} can challenge the proposed scheme with an *T* advantage, an actor *Q* can be created to play the DBDH game with *T*/2 advantage. Then to determine *W*, if *W* = *m*(*h*,*h*)^*stw*^, *Q* generate 1, otherwise, 0. The proof is further explained in five steps as follows:

**Step-1:** ***A*** chooses *AS*′ and a challenge attribute set *U*′ and then submit it to *Q*, such that ∃ *a*’ ∈ *U*’ that satisfies *U*’ to *AS*′.
**Step-2:** *Q* chooses the random values η,*ρ* ∈ ℤ_*p*_ and assign the following parameters: *h*_1_ = *h*^ε^, *h*_2_ = *h*^*s*^, $\varphi_{1}=h^{\eta }\prod_{a_{i\in AS^{\prime }}}h_{1}^{-a_{i}^{\prime }}$, $\varphi_{2}=h^{\rho }\cdot h_{1}^{-1}$, *Z* = *m*(*h*_2_,*h*_1_) = *m*(*h*,*h*)^*st*^. Then *Q* generates *K*_*pub*_ = (*φ*_1_,*φ*_2_,*h*,*h*_2_,*h*_1_,*Z*) and then submit it to ***A***.
**Step-3:** ***A*** ensures if *U* ≠ *AS*’, *U*’ ≠ *U*, otherwise, *Q* terminates the operation and randomly assumes the values. Thus, *Q* computes $K_{prv}^{U^{\prime }}$ as follows:
  - *Q* chooses a random value δ′ ∈ ℤ_*p*_, and generate $E_{1}^{\prime }=h^{\delta^{\prime }}\cdot h^{\frac{-1}{U-U^{\prime }}}$, $E_{2}^{\prime }=h_{2}^{\frac{-\eta }{U-U^{\prime }}}\cdot {\left(\varphi_{1},\prod_{a_{j\in U}}h_{1}^{a_{j}}\right)}^{\delta^{\prime }}$.
  - *Q*, then, submit the $K_{prv}^{U^{\prime }}=\left\{E_{1}^{\prime },E_{2}^{\prime }\right\}$ to ***A***.

From a, b above, if $\delta =\delta^{\prime }-\frac{t}{U-U^{\prime }}$, then, $K_{prv}^{U^{\prime }}$ is valid. That is:

$$E_{1}^{\prime }=h^{\delta^{\prime }}\cdot h_{2}^{\frac{-1}{U-U^{\prime }}}=h^{\delta^{\prime }-\frac{t}{U-U^{\prime }}}=h^{\delta }$$ (12)

$$E_{2}^{\prime }=h_{2}^{\frac{-\eta }{U-U^{\prime }}}\cdot {\left(\varphi_{1},\prod_{a_{j\in U}}h_{1}^{a_{j}}\right)}^{\delta^{\prime }}$$ (13)

Thus, by multiplying the value of $E_{2}^{\prime }$ with (*h*^*st*^∙*h*^-*st)*^,

$$E_{2}^{\prime }=h_{2}^{s}\cdot {\left(\varphi_{1},\prod_{a_{j\in U}}h_{1}^{a_{j}}\right)}^{\delta }$$ (14)

**Step-4:** With this, ***A*** submits the data messages Ð′ and Ð′′ to *Q* whom then tosses a fair binary coin *ω*∈{0,1}, then, creates ${\text{TK}}^{\prime }=\left({Ð}_{\omega },h^{w},W,{\left(h^{w}\right)}^{\eta },{\left(h^{w}\right)}^{\rho }\right)$. If *W* = *m*(*h*,*h*)^*stw*^, then TK’ is a valid encrypted text. If *W* is a random value in ***G***′ then ***A*** will assume that *TK*’ and *ω* are two separate values.
**Step-5:** As *Q* act precisely as expected in Step-3, thus ***A*** performs a guess *ω*’. *Q* generate, 1 as its output only if *ω*’ = *ω*. Hence, the probability *P* of thwarting the DBDH challenge can be deduced as follow:

$$P\left[\omega^{\prime }=\omega \right]-1/2=P\left[\omega^{\prime }=\omega |\omega =0\right]\cdot P[\omega =0]+P\left[\omega^{\prime }=\omega |\omega =1\right]\cdot P[\omega =1]-1/2=T/2$$ (15)

## Experimental results and evaluation

This section delves into the simulation environment and the performance metrics employed in the development and assessment of the proposed model. The section outlines the framework within which the model was tested, highlighting the technical parameters and methodologies that underpin the simulation process. The evaluation of the proposed model is critically examined in comparison with existing models, specifically those introduced by H.R. Hasan et al. [21] and H. Saidi et al. (DSMAC) [57], as referenced in the study. The rationale for selecting these particular models for comparison is grounded in their relevance and the feasibility of comparing their performance and outcomes with those of the proposed model within similar operational contexts.

To clarify the advancements in security and operational efficiency achieved by the proposed model, a detailed comparative security and operational analysis is provided. This analysis not only benchmarks the proposed model against the aforementioned models but also highlights the innovative aspects of security enhancements and operational efficiencies it introduces. By drawing these comparisons, the analysis aims to underscore the contributions of the proposed model to the field and its potential to address existing gaps in the literature.

Furthermore, this section also presents a reflective discussion on the limitations encountered during the study and proposes avenues for future research and improvements. Acknowledging the limitations provides a balanced view of the proposed model’s capabilities and areas where further enhancements are needed. It sets the stage for subsequent research efforts to build on the foundation laid by this study, aiming for advancements that could address the identified limitations and potentially introduce new features or optimizations. This forward-looking perspective is crucial for the continuous evolution of the model and its applicability to real-world scenarios.

### Design and implementation of smart contracts in PatCen

The PatCen system designs and executes smart contracts to automate and robustly enforce data access rules in a secure and transparent manner within the blockchain framework. Solidity, a specialized high-level programming language, implements these contracts on the Ethereum blockchain. The following is a comprehensive examination of the design logic, inherent self-triggering characteristics, and execution procedure of these smart contracts.

*Design Logic and Self-Triggering Mechanism*: PatCen specifically engineers the smart contracts to manage a range of functions, such as data access requests, permission assessment, and transaction recording on the blockchain. The fundamental principle of these contracts is to guarantee that only specifically authorized users may retrieve confidential healthcare information, in accordance with their predetermined responsibilities and permissions. The contracts offer self-triggering capabilities, designed to initiate automatically when specific conditions, like a user’s submission of an access request, are satisfied.

*Execution within the Blockchain Environment*: When smart contracts are deployed on the Ethereum blockchain, they undergo decentralized execution. Following a user’s submission of a transaction, such as an access request, the relevant smart contract autonomously executes the request in accordance with the predetermined logic. The contract authenticates the user’s role and assesses if the request satisfies the specified criteria for access. If the specified requirements are satisfied, the contract records the transaction on the blockchain, ensuring the transparency and immutability of all actions.

### Stack of technology in PatCen

The PatCen system employs a robust technological stack that enables the blockchain-based data management framework to function securely and effectively. The following is a comprehensive summary of the primary technologies employed:

*Ethereum blockchain*: PatCen is based on the Ethereum blockchain, a well-known and established system that provides strong smart contract functionality. The permissioned network architecture of Ethereum is well-suited for healthcare applications, where ensuring data security and implementing limited access are of utmost importance. The inherent decentralized structure of the blockchain guarantees the safe and immutable recording of all transactions and data access events, thus establishing a robust foundation of trust and accountability.

*Solidity*: Solidity serves as the primary programming language for creating the PatCen smart contracts. The programming language was purposefully developed to facilitate the creation of contracts that are executed on the Ethereum Virtual Machine (EVM). The syntax of Solidity bears resemblance to that of JavaScript, making it easily comprehensible for developers while nevertheless offering robust functionalities for implementing complex logic within smart contracts. The selection of Solidity guarantees that the contracts exhibit both efficiency and security, using inherent measures to mitigate prevalent vulnerabilities.

*Metamask*: Metamask serves as a software tool to manage Ethereum accounts and streamline interactions with the public blockchain. The technology functions as an intermediary between the user’s web browser and the Ethereum network, facilitating the safe management of private keys, transaction signing, and interaction with the PatCen system. The integration of Metamask facilitates convenient system access for users, enabling them to safeguard their credentials and data while retaining appropriate control.

*Web3*.*js*: The Web3.js library is a JavaScript framework that enables seamless communication between the front-end application and the Ethereum blockchain. This functionality enables the PatCen system to execute transactions, engage with smart contracts, and extract data from the blockchain. The Web3.js framework facilitates a smooth connection between the user interface and the blockchain backend, hence allowing instantaneous updates and interactivity inside the decentralized ecosystem.

### Implementation setup

In the development of the proposed model, we utilized Python and Solidity programming languages for their robustness and compatibility with blockchain technologies. The deployment and storage of Ethereum were facilitated through the Metamask Ethereum client, showcasing the practical application of the model within the Ethereum blockchain environment. Our experimental setup was conducted on the Rinkeby testnet, employing a Proof of Authority (POA) consensus mechanism to validate transactions efficiently and securely, which is critical for maintaining the integrity and reliability of the blockchain operations.

The construction of the model’s logic and the interactions with smart contracts were orchestrated using the Web3.py Python library alongside other relevant modules. This approach enabled precise and efficient communication between the model and the Ethereum blockchain, ensuring seamless execution of operations within the blockchain environment.

The experimental simulations were carried out on personal computers equipped with an AMD PRO A8-9600B R5, 10 COMPUTE CORES 4C+6G, operating at 2.40 GHz, providing a stable and controlled environment for conducting the tests. This hardware setup was selected to ensure a balance between performance and accessibility, allowing the experiments to be replicable in environments accessible to most researchers.

In conducting these experiments, it was paramount to maintain a consistent setup across all test scenarios to avoid bias and ensure the reliability of our observations and results. By implementing the models in identical setup scenarios, we aimed to achieve the most accurate and unbiased comparison of their performances, thereby providing a solid foundation for evaluating the effectiveness and efficiency of the proposed model in a blockchain environment.

### Experimental evaluations

In evaluating the performance and quality of any system, specific metrics are essential for providing a comprehensive assessment. Similarly, for the proposed system, a set of key performance indicators has been established to offer a holistic view of its efficiency and effectiveness. These metrics include:

- Cost Analysis: This metric examines the system’s economic efficiency, focusing on gas consumption and associated gas prices. Understanding the financial implications of operating the system within a blockchain environment, where transactions and operations incur costs in the form of gas fees, is critical.
- Encryption and Decryption Times: These metrics assess the efficiency of the cryptographic processes within the system. The speed at which data can be securely encrypted and subsequently decrypted is vital for evaluating the system’s performance in protecting sensitive information while ensuring accessibility for authorized users.
- Key Generation Time: The time required to generate secure cryptographic keys is another critical performance indicator. This metric provides insights into the efficiency of the system’s security mechanisms, specifically in the context of generating robust keys that underpin the overall security of the cryptographic processes.
- Computational Latencies: This metric measures the delay or latency in processing operations within the system. Lower computational latencies are indicative of a more responsive and efficient system, which is especially important in applications requiring real-time or near-real-time processing capabilities.
- Computational Throughput: This metric evaluates the system’s ability to process a high volume of operations within a given time frame. Higher computational throughput indicates a system’s capacity to handle larger loads efficiently, making it a crucial metric for assessing scalability and performance under varying conditions.

Together, these metrics provide a comprehensive framework for evaluating the proposed system’s performance, offering insights into its operational efficiency, security, and cost-effectiveness. This approach ensures a balanced evaluation, taking into account both the technical and economic aspects of the system’s operation.

### Cost analysis

The Remix environment represents a pioneering method for meticulously documenting the financial intricacies of transactions within the Ethereum network. In this innovative approach, every log entry comprehensively encapsulates the expenses associated with transactions and executions. A crucial aspect of navigating this ecosystem is understanding the transaction speeds, which are intricately tied to gas pricing, predominantly denoted in Gwei (gas price). Smart contract development necessitates a meticulous assessment of gas costs to preempt any potential ancillary expenditures. It’s worth noting that miners tend to prioritize transactions with higher Gwei values, thus enhancing the likelihood of processing transactions with elevated Gwei prices.

Various factors, including loops, arrays, mappings, variable storage, and data types, profoundly influence transaction costs. The paramount concern lies in the practicality and efficiency of the solution. As such, our proposed methodology capitalizes on the inherent immutability of the blockchain, leveraging events and logs instead of on-chain storage.

While the price of gas may fluctuate depending on the day and time of year, our methodology has been rigorously evaluated during non-peak periods, specifically in June 2021, when the costs associated with executing and transacting operations are relatively lower. On June 25, 2021, the ETH Gas Station recorded gas prices of 15.1, 15.1, 5.7, and 5.7 Gwei for the fastest, fast, average, and lowest categories, respectively. Table 3 provides a detailed cost analysis in United States dollars (USD) based on the average gas price of 5.7 Gwei.

**Table 3:** Cost analysis table.

Function name	Transaction Cost	Execution Cost	Cost USD
*addpatient*	22409	2391	$0.1325
*adduser*	31357	3849	$0.1855
*grantaccess*	23549	2297	$0.1375
*deleteuser*	20349	2033	$0.121
*deletepatient*	20702	2067	$0.121

Average cost: $ 0.1395

Table 3 elucidates the transaction costs and execution expenses denominated in Gwei. Notably, the adduser function incurs a maximum cost of $0.1855. While this price is considered modest, it emerges as the most expensive among all functions within our proposed system. The adduser function serves a pivotal role in facilitating user addition and classification based on their responsibilities within the system. Despite its relatively higher cost, the absence of loops or arrays in the methods suggests that the anticipated expenses remain inconsequential.

**Fig 3:**
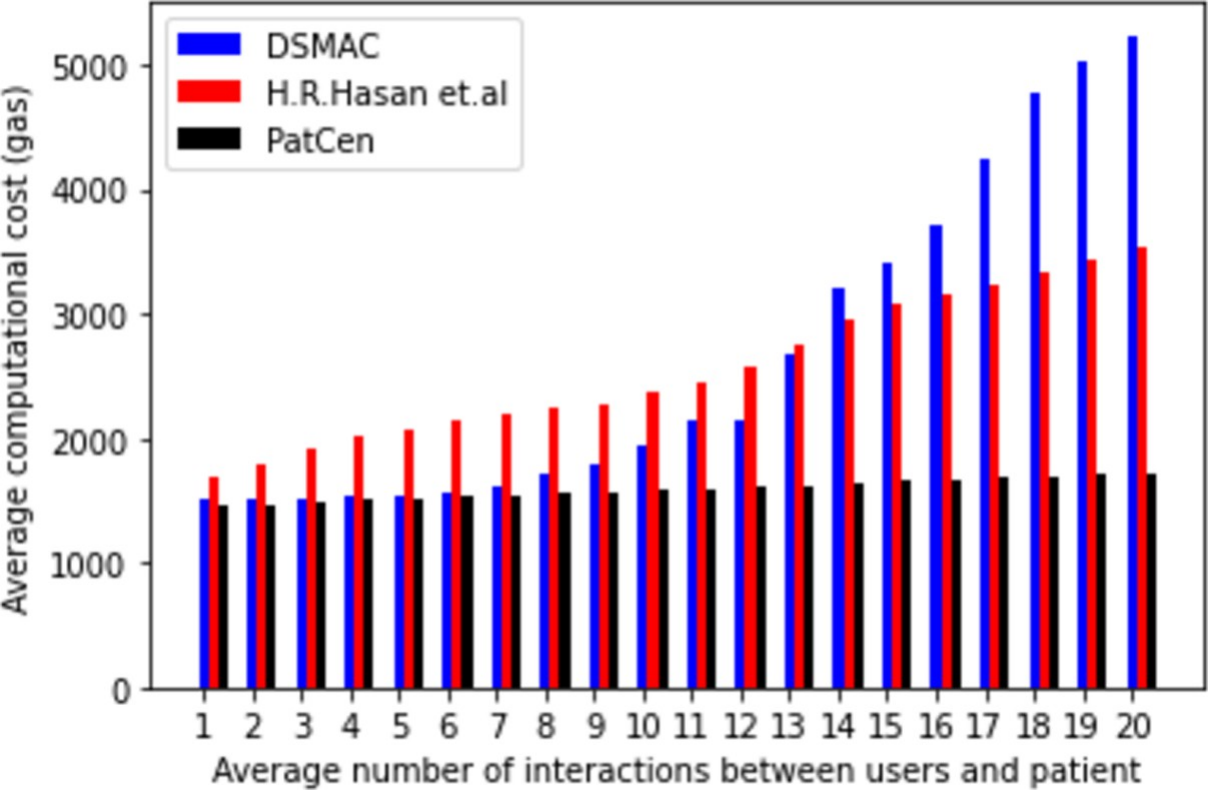
Average computational cost (execution and transaction costs).

In comparison, our proposed approach boasts an average cost of $0.1395 per full program execution, while the benchmark model [21] demonstrates an average cost of $0.16375, based on the gas price at ETH Gas Station on June 25, 2021. The results shown in Fig 3 show that our proposed approach cuts computing costs by a large amount compared to the average execution and transaction costs in the benchmark models. These margin disparities highlight our methodology’s efficacy and cost-efficiency.

In terms of gas consumption per access request and grant, Fig 3 shows that the proposed model is much less sensitive to more interactions with third-party users than benchmark models. The deliberate avoidance of the clustering approach, which demands extensive computational efforts in smart contract development, accounts for this. Furthermore, our model demonstrates a decrease in transaction overhead as third-party users increase, leading to a notable reduction of around 46 percent in total block time validation, as reported by Etherscan.

### Encryption, decryption & key generation latencies

The core concept hinges on the interaction between a myriad of third-party users and the retrieval of specific patient records within the system. This model delves into an average of twenty (20) interactions between third-party users and patients to scrutinize their dynamics. Central to the model’s operation is the access structure, where an upsurge in third-party user requests corresponds to a proportional increase in the system’s time allocation. Our study quantifies the average time overhead across three key performance metrics—encryption, decryption, and key generation—analyzing them individually based on the frequency of interactions with third-party users within the system.

**Fig 4:**
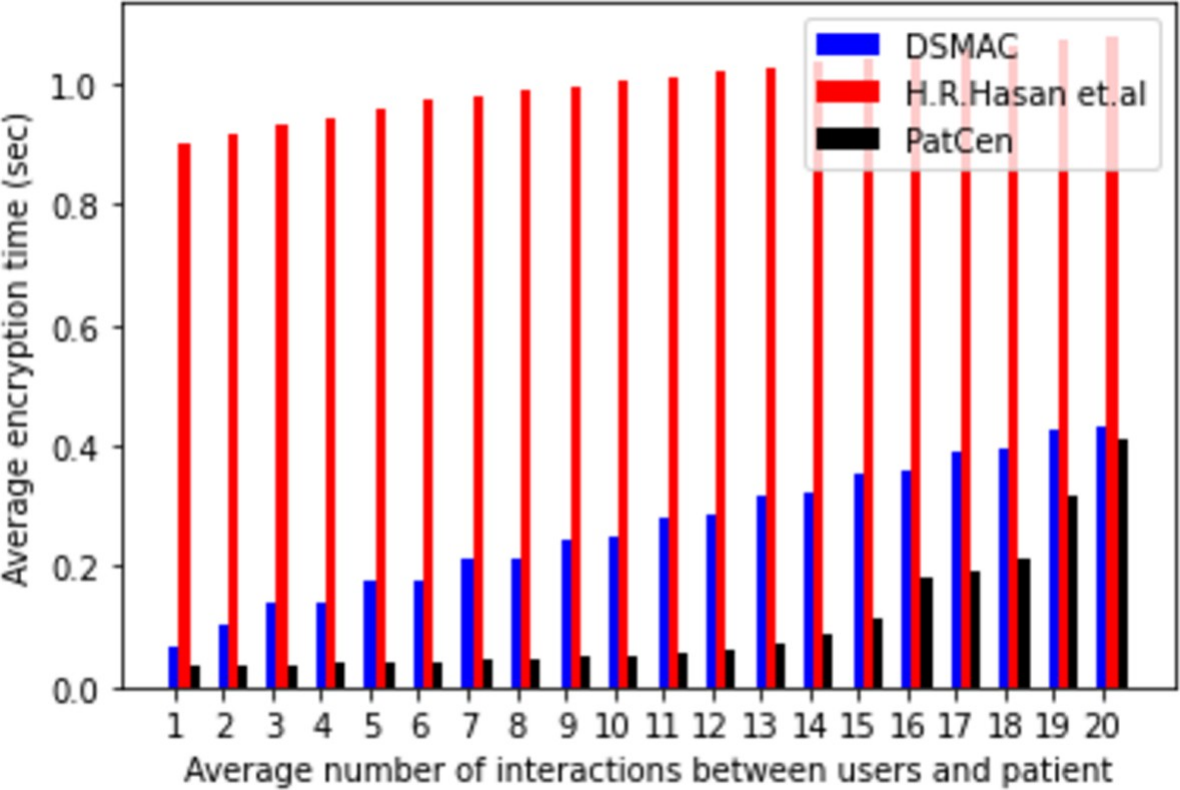
Average encryption time.

**Fig 5:**
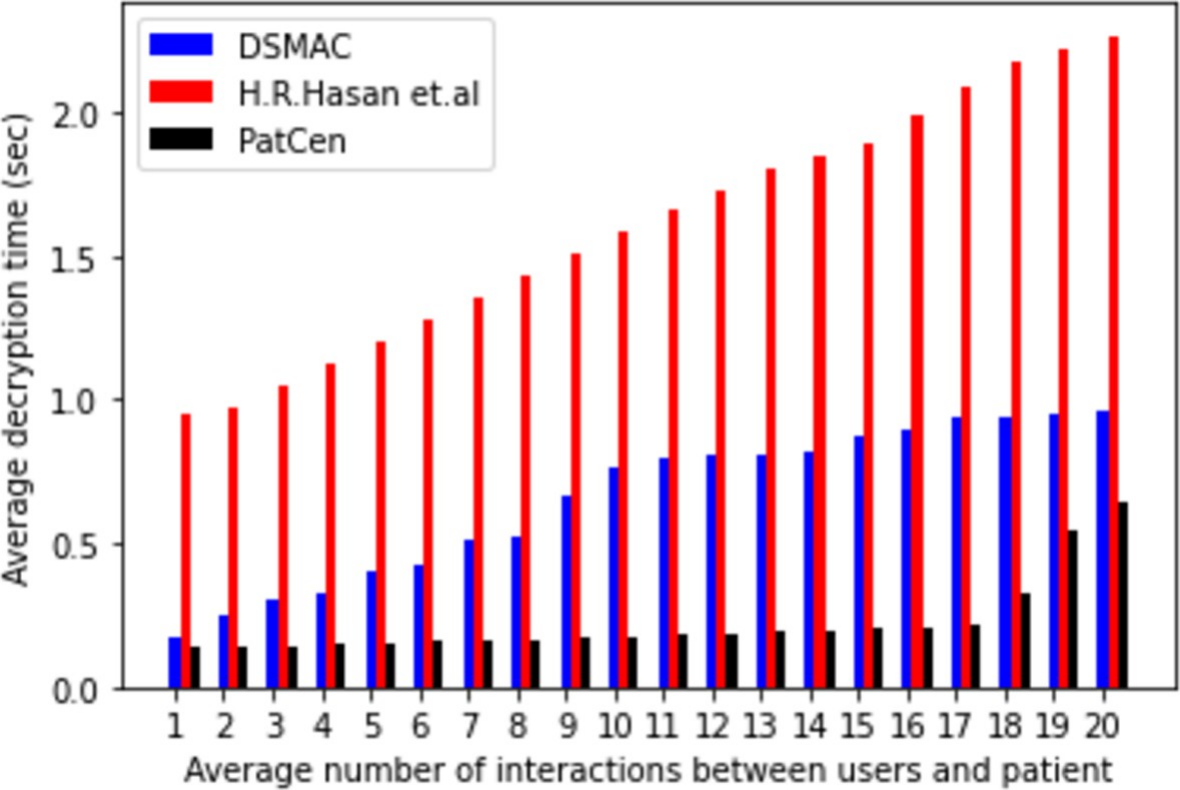
Average decryption time.

**Fig 6:**
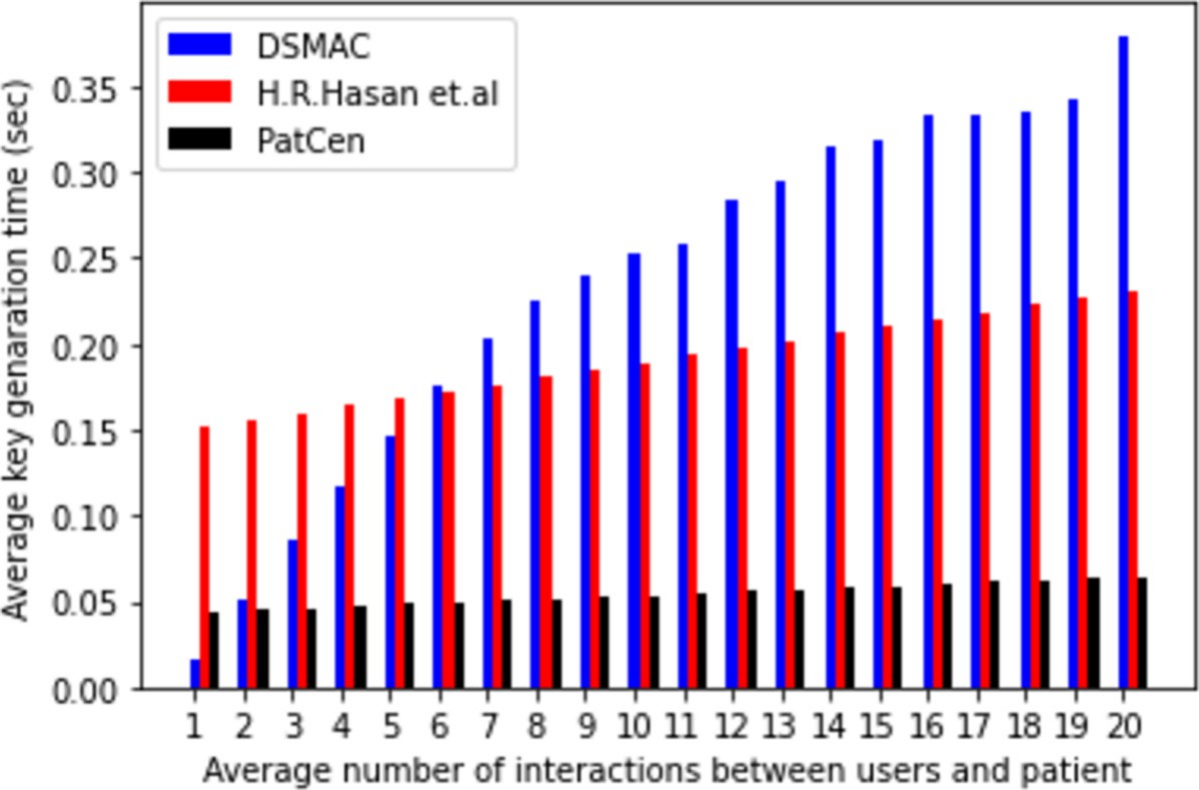
Average key generation time.

The system intricately links temporal costs to the volume of interactions with third-party users. Figs 4–6 delineate the correlation between the model’s encryption, decryption, and key generation time, and the frequency of interactions with third-party users. Notably, as the number of interactions escalates, so does the time required for encryption, decryption, and key creation. Our proposed framework demonstrates average times of 0.036805556, 0.050916667, and 0.062111111 for encryption, decryption, and key generation, respectively. In contrast, the model by H.R. Hasan et al. exhibits times of 0.416, 0.2825, and 0.227 for the same operations, while the DSMAC model showcases times of 0.263629408, 0.65823956, and 0.235203387, respectively.

Moreover, our findings underscore a positive correlation between time passage and the number of interactions across all models. However, the proposed model evinces a minimal and consistent rise in time compared to both base models, particularly under heightened interaction volumes. Conversely, the benchmark models exhibit a substantial increase in overhead as interactions surge. Notably, the proposed model’s overhead remains consistently low and stable under moderate interaction volumes, a stark contrast to the benchmark models.

The volatility observed in the benchmarks is attributed to the utilization of POW consensus and the implementation of extensive encryption and decryption processes by the central administration. In contrast, our proposed model employs the POA consensus mechanism, which contributes to its enhanced efficiency in access control. Based on our analysis of computational overhead and time duration, it is evident that our proposed model outperforms benchmark models in terms of both access request and grant time for patient test data, showcasing a higher level of efficiency.

### Computational latency and throughput

Computational latency, in this context, signifies the time elapsed from the submission of a transaction by a user to its processing and recording in the ledger. The meticulous monitoring, documentation, and comparison of computational latency serve to gauge its performance relative to benchmark models. Our assessment of transaction latency performance employed a background timer program operating concurrently with transaction executions, with time measurement determined by the system processor clock, contingent upon the prevailing processor schedule.

**Fig 7:**
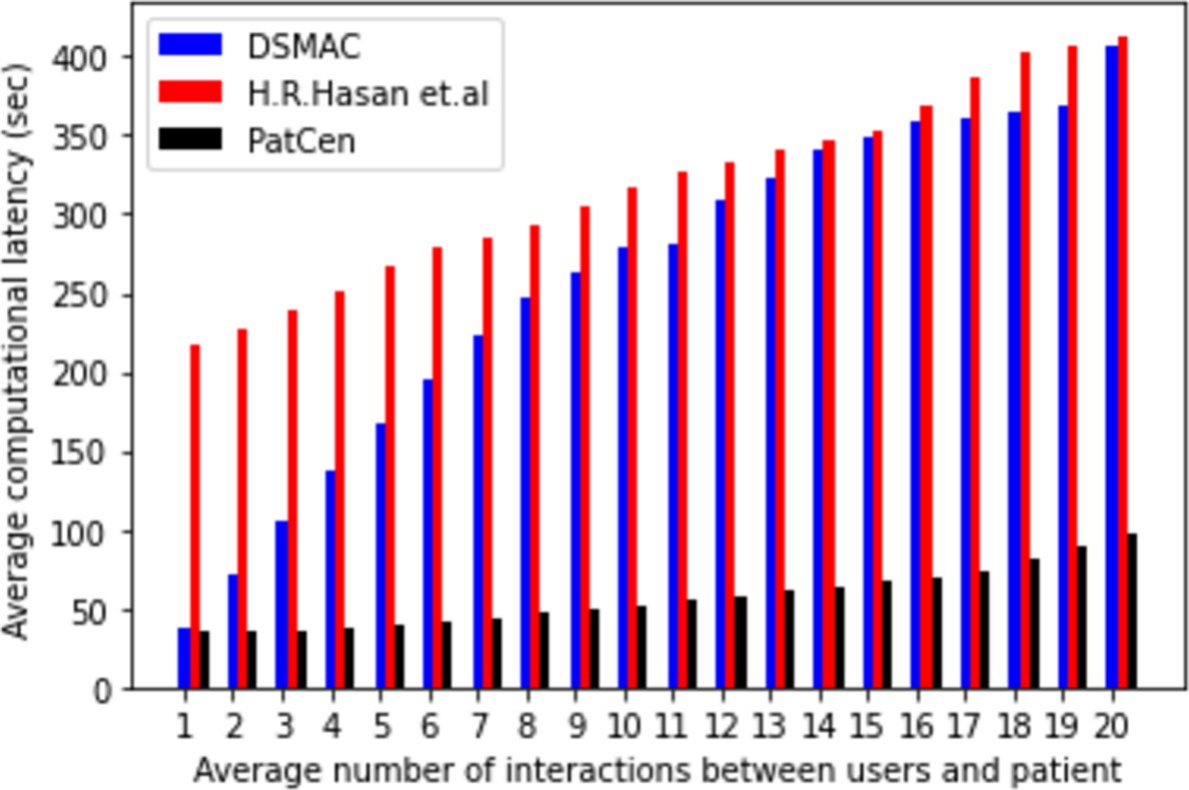
Average computational latency.

The model we propose demonstrates reduced computational latency, as evidenced by the data in Fig 7, compared to benchmark models. A consistent increase in average computational latency is observed with the escalation of users’ interactions across all models. However, the average latency exhibited by our proposed PatCen model is notably reduced compared to that seen in the benchmark models. This reduced delay can be attributed to the implementation of the proof-of-authority consensus mechanism. It is noteworthy that there exists an inverse relationship between security level and latency, whereby higher security is associated with lower latency.

The PatCen model has the potential for a significant influx of access requests, primarily due to the diverse range of interactions with third-party users that require management and processing. Computational throughput was evaluated by measuring computing overhead per unit of gas consumption during the progressive increase in user interactions. Subsequently, we compared the average computational throughput of our proposed model with that of benchmark models.

Initially, both the proposed scheme and the DSMAC model exhibit comparable throughputs. However, the extensive encryption and decryption procedures conducted by the central administration in the DSMAC and H.R. Hasan et al. methods throughout various stages of credential verification and query processing consistently alter the ledger’s state, resulting in lower throughput compared to the PatCen model.

**Fig 8:**
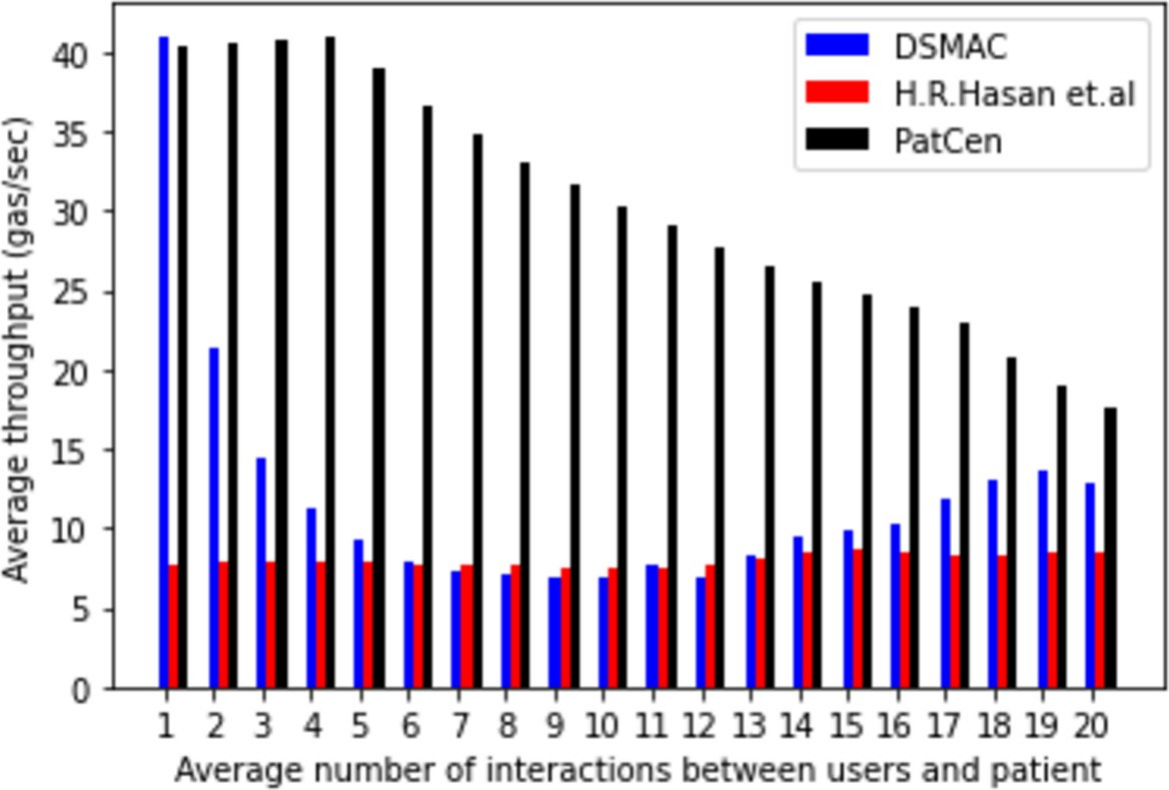
Average computational throughput.

Fig 8 illustrates a significant decline in the throughput of both DSMAC and H.R. Hasan et al. models as the number of users’ interactions grows. Therefore, we can deduce that the proposed PatCen model outperforms benchmark models in terms of effectiveness and efficiency.

### Comparative security and operational analysis

Table 4 provides a comprehensive comparative analysis of the security and operational aspects between the proposed PatCen protocol and two existing schemes developed by H.R. Hasan et al. [21] and H. Saidi et al. (DSMAC) [57]. The assessment categorized system performance using "Yes" and "No" indicators to signify the extent to which each system fulfilled the evaluated security and operational criteria.

**Table 4:** Security and operational comparison.

Security Features	DSMAC [57]	H.R. Hasan et al. [21]	PatCen
User registration	Yes	Yes	Yes
User validation	Yes	No	Yes
Session key security	No	No	Yes
Considering patient privileges	Yes	Yes	Yes
Fine grain data access	No	No	Yes
User anonymity	No	No	Yes
Patient privacy	Yes	No	Yes

The analysis reveals that the systems presented in references [21, 57] lack session key security and fine-grained data access during data transfers, as highlighted in the table. Specifically, the benchmark systems [21, 57] failed to ensure the confidentiality of third-party users, particularly concerning user anonymity within the system. Furthermore, this study shows that the methods used in the benchmark systems have higher computing overhead, lower throughput, and higher latencies, thereby falling short in delivering enhanced security and efficiency within the system.

In contrast, the PatCen model successfully satisfied all security and operational requirements considered in this study. This underscores the efficacy and robustness of the proposed model in meeting the stringent demands of security and operational excellence within the system.

### Justification for the superior performance of the PatCen model

The superior performance of the PatCen model compared to existing methods can be attributed to several key innovations and optimizations that directly address the unique challenges associated with managing infectious disease test records in a healthcare setting. These advancements ensure that PatCen not only meets but exceeds the requirements for security, efficiency, and scalability, which are critical in this context.

### Granular data access control

The PatCen model employs a granular data access control mechanism that is more advanced than the traditional role-based access controls used in other methods. This mechanism allows for fine-tuned permissions, enabling patients to precisely manage who can access their data and under what circumstances. This level of control is essential in healthcare, where data privacy and patient autonomy are paramount.

- **Impact:** The enhanced control over data access reduces the risk of unauthorized access and ensures that only necessary information is shared with third parties, improving both security and compliance with privacy regulations such as GDPR and HIPAA. Other methods, which often use broader access control schemes, may not provide the same level of protection or flexibility, leading to potential privacy risks and inefficiencies.

### Optimized blockchain framework with PoA consensus

PatCen’s use of a permissioned blockchain with a Proof-of-Authority (PoA) consensus mechanism is a significant improvement over more traditional consensus methods like Proof-of-Work (PoW) or Proof-of-Stake (PoS). The PoA mechanism is specifically designed for environments where the network participants are known and trusted, which is typical in healthcare networks.

- **Impact:** This approach significantly reduces computational overhead and enhances transaction speed, making the system more efficient and scalable. In contrast, other models that rely on PoW or PoS may experience higher costs and slower performance, particularly in large-scale implementations, which can limit their practical applicability in real-time healthcare scenarios.

### Game-theoretic security enhancements

A distinctive feature of the PatCen model is its integration of game theory to enhance security. By modelling the interactions between the system and potential adversaries, PatCen can anticipate and mitigate security threats more effectively than traditional static security measures.

- **Impact:** This proactive security strategy ensures that the system remains resilient against evolving threats, providing a higher level of data protection. Other methods, which may not incorporate such dynamic security approaches, could be more vulnerable to new types of attacks, leading to potential data breaches and compromised patient information.

### Scalability and real-time performance

The PatCen model is designed with scalability in mind, ensuring that it can handle a growing number of users and transactions without a decline in performance. The system’s architecture supports high throughput and low latency, which are critical for applications that require real-time access to healthcare data, such as during emergency medical situations or when rapid verification of test results is needed for travel.

- **Impact:** The ability to maintain performance as the system scales is a major advantage over other methods that may struggle with increased load, leading to bottlenecks and slower response times. This makes PatCen more suitable for widespread deployment in diverse healthcare environments.

In conclusion, the PatCen model’s superior performance is the result of its innovative approach to data access control, optimized blockchain framework, advanced security measures, and scalability. These features directly address the limitations of existing methods, making PatCen a more effective and reliable solution for managing infectious disease test records in healthcare settings. By overcoming the drawbacks of traditional models, PatCen ensures that healthcare data is managed securely, efficiently, and in a manner that respects patient autonomy and privacy.

## Discussions

In this section, we provide an in-depth examination of the PatCen model, exploring its underlying mechanisms, compliance with ethical and regulatory standards, and the potential limitations and future directions of the system. We begin by discussing the PoA consensus mechanism and its role in ensuring the efficiency, security, and performance of the PatCen blockchain. Following this, we address how the model aligns with critical regulatory frameworks such as GDPR and HIPAA, highlighting the specific measures implemented to protect patient data and ensure compliance with legal requirements. Additionally, we evaluate the limitations of the proposed model, particularly in relation to scalability and the reliance on the Ethereum blockchain, and outline our vision for future improvements. This discussion provides a holistic view of the strengths and areas for enhancement within the PatCen system, setting the stage for ongoing research and development in the field of blockchain-based healthcare solutions.

### Proof-of-Authority (PoA) consensus mechanism in PatCen

The PoA consensus method is an essential element of the PatCen system, guaranteeing efficient and safe functioning within our permissioned blockchain framework. PoA is particularly well-suited for healthcare environments because the network members, such as hospitals, labs, and healthcare providers, are established and reliable institutions. This technique provides several benefits that are well-suited to the needs of handling confidential health information.

1. Efficiency and Reduced Processing Demands: In contrast to PoW or PoS systems that require substantial processing resources and energy use, PoA functions effectively with minimum computational expenses. Validators in a PoA system are pre-approved and recognized by the network, which means there is no need for complex cryptographic puzzles or large stake holdings. To maintain low operating expenses, healthcare settings require rapid and reliable data processing efficiency.
2. Centralized Control with Identified Validators: In a PoA blockchain, a trusted select few validators are responsible for confirming transactions and appending them to the blockchain. These validators are typically organizations or individuals that have a recognized authority within the network, such as healthcare institutions or regulatory authorities. The centralized management of the system strengthens security by guaranteeing that only authorized and reputable entities have the authority to verify transactions. Additionally, it lessens the probability of malicious behaviour by holding validators accountable for their actions and enabling easy detection and auditing.
3. Contribution to Security and Performance: The PoA mechanism plays an important role in enhancing the PatCen system’s security and functionality. The system mitigates the danger of unauthorized access or tampering with patient data by imposing restrictions on the number of validators and confirming their credibility. In addition, the efficient validation procedure allows for quicker transaction times and increased throughput, guaranteeing real-time accessibility to healthcare data, which is vital in times of public health crises.

Overall, the PoA consensus process is very suitable for the specific requirements of a healthcare-oriented blockchain such as PatCen. It offers a harmonious combination of efficiency, security, and performance, making it a perfect option for handling the delicate and time-sensitive data that is inherent in healthcare systems.

### Alignment with ethical standards and regulatory frameworks

The PatCen system specifically adheres to the General Data Protection Regulation (GDPR) and the Health Insurance Portability and Accountability Act (HIPAA) to ensure compliance with ethical standards and regulatory frameworks, which are essential for safeguarding healthcare data from unauthorized access and misuse. The implementation of this alignment guarantees that the system not only safeguards patient confidentiality but also adheres to legal obligations pertaining to the management of personal health data.

#### Adherence to the GDPR

The GDPR is an all-encompassing regulatory framework that oversees the handling of personal data within the European Union. PatCen’s design aligns with the GDPR’s fundamental principles, namely data minimization, obtaining permission, and the right to access and erase personal data.

Data Minimization: PatCen implements stringent measures to ensure that only the essential data is collected and processed. The system implements a granular access control mechanism that restricts access to specific data characteristics based on the user’s role and the request’s context. This methodology effectively mitigates the potential vulnerability of sensitive information, thereby reducing the likelihood of illegal intrusion.

Consent Management: The system integrates procedures to acquire and oversee patient permission prior to engaging in any data processing operations. The GDPR outlines explicit and informed permission mandates that allow patients to revoke their authorization at any given moment.

Right to Access and Erasure: PatCen protects patients’ legal rights to access their data and request its deletion. Patients can access their records and configure access rights through the system’s user interface. In the event that a patient communicates a desire for data deletion, the system guarantees the secure erasure of all pertinent data from the blockchain, upholding the "right to be forgotten" as mandated by the GDPR.

#### Adherence to HIPAA

HIPAA establishes the minimum requirements for safeguarding confidential patient information inside the United States. We have developed the PatCen system to incorporate functionalities that align with the data privacy and security regulations outlined by HIPAA. These features specifically focus on ensuring the protection of electronic protected health information (ePHI).

Data Security: PatCen employs strong encryption techniques to protect ePHI during storage and transmission. This measure ensures the preservation of health information confidentiality and security, thereby reducing the risk of unauthorized access and breaches. The use of blockchain technology serves to augment security measures by providing an unalterable log of all data transfers, a crucial aspect for ensuring auditability and adherence to the security regulations set out by HIPAA.

Access Control: PatCen’s access control measures guarantee that only those with permission may access ePHI, in compliance with HIPAA’s privacy guidelines. The enforcement of role-based access restrictions inside the system is facilitated by smart contracts, which dynamically configure permissions according to the user’s role and the particular data they are authorized to access.

Auditability: In order to comply with HIPAA’s standards for auditing and accountability, PatCen offers a thorough audit trail of all data access and change activities. The inherent characteristics of transparency and immutability in blockchain technology guarantee the recording of all operations and prevent any alterations, thus establishing a reliable framework for monitoring and tracing data handling activities.

In conclusion, PatCen ensures the appropriate handling of sensitive healthcare data by adhering to GDPR and HIPAA, thereby upholding patient privacy and complying with international regulatory requirements. The design of the system places significant emphasis on the elements of data security, consent management, and secure access, establishing it as a resilient solution for the administration of health information in a manner that adheres to legal requirements and ethical principles.

### Limitations and future improvements

In the healthcare sector, the integration of blockchain technology is steadily gaining traction owing to its prowess in enhancing data security, integrity, and accessibility. This paper introduces the PatCen model, a secure framework tailored for implementing granular access control for infectious disease test information. Leveraging blockchain technology, the PatCen concept fortifies security measures. Its decentralized architecture safeguards against tampering or illicit modifications, thereby upholding the integrity of critical health data. Transparency among all participants in the blockchain network is ensured, while access control mechanisms facilitate secure data sharing with authorized entities. Furthermore, the utilization of smart contracts and distributed consensus techniques fosters trust among stakeholders in the healthcare domain.

However, the model’s effectiveness in expansive and dynamic environments may be limited by its constrained experimental framework and reliance on the Ethereum blockchain for storage and processing. The current state and future advancements of the Ethereum network could impact the scalability and efficacy of the approach. Consequently, our future endeavors will prioritize scalability as a primary objective while simultaneously safeguarding both patient and user privacy rights.

Future investigations will broaden the scope of the model to encompass various forms of access-related threats. Researching deeper into anonymity and privacy, as pivotal components, will be imperative to adeptly address any unforeseen challenges that may arise. Moreover, further exploration will be conducted to evaluate the model’s potential for application in tangible devices. Additionally, forthcoming work will provide detailed insights to enhance understanding, including specific features of the mobile application and the user interface for granting access. These components collectively empower patients to confer privileges upon other users.

## Conclusion

The objective of this study is to analyze the conceptualization, implementation, and assessment of a blockchain-driven access control mechanism for electronic health records. The proposed system facilitates the generation of records for tests linked to infectious diseases, the storage of patient data, and the implementation of access restrictions to ensure that only authorized users, based on their respective roles, may access the data. In this study, both the data and the session key are encrypted to ensure that unauthorized users are unable to access the data by abusing the session key of another user’s computer. The proposed solution underwent an evaluation of its methodologies, which included a full cost analysis, encryption and decryption processes, key production, as well as measures of latencies and throughput. The results of the security study indicate that the proposed solution demonstrates safety within the framework of the DBDH game theory and successfully attains session key security according to the ROR formal security analysis game theory, among other notable results. The simulation results demonstrate that the proposed approach exhibits reduced computational cost, increased throughput, and decreased latencies in comparison to the benchmark models. The cryptographic simulation results demonstrate that the proposed model exhibits significantly enhanced security and efficiency compared to the benchmark model. The proposed approach aims to mitigate the spread of infectious diseases by effectively collecting, managing, and securely disseminating time-sensitive data to authorized users with the explicit consent of the patients involved.
